# Dose-finding study of intensive weekly alternating schedule of docetaxel, 5-fluorouracil, and oxaliplatin, FD/FOx regimen, in metastatic gastric cancer

**DOI:** 10.18632/oncotarget.24861

**Published:** 2018-04-17

**Authors:** Gemma Bruera, Silvia Massacese, Antonio Galvano, Antonella Dal Mas, Stefano Guadagni, Giuseppe Calvisi, Eugenio Ciacco, Antonio Russo, Enrico Ricevuto, on behalf of Oncology Network ASL1 Abruzzo, Italy

**Affiliations:** ^1^ Oncology Territorial Care, S. Salvatore Hospital, Oncology Network ASL1 Abruzzo, University of L'Aquila, L'Aquila, Italy; ^2^ Department of Biotechnological and Applied Clinical Sciences, University of L’Aquila, L’Aquila, Italy; ^3^ Pharmacy Unit, S. Salvatore Hospital, Oncology Network ASL1 Abruzzo, L’Aquila, Italy; ^4^ Medical Oncology, Department of Surgical, Oncological and Stomatological Sciences, University of Palermo, Palermo, Italy; ^5^ Pathology, S. Salvatore Hospital, Oncology Network ASL1 Abruzzo, L'Aquila, Italy; ^6^ University General Surgery, S. Salvatore Hospital, Oncology Network ASL1 Abruzzo, University of L'Aquila, L'Aquila, Italy

**Keywords:** docetaxel, 5-fluorouracil, and oxaliplatin, FD/FOx intensive regimen, dose-finding study, first-line triplet chemotherapy, metastatic gastric cancer

## Abstract

**Introduction:**

Proper administration timing, dose-intensity, efficacy/toxicity ratio of triplet docetaxel (DTX), 5-fluorouracil (5-FU), and oxaliplatin (OXP) should be improved to safely perform three-drugs intensive first line in advanced gastric cancer (GC). This dose-finding study investigated recommended 5-FU and OXP doses, safety of triplet regimen and preliminary activity.

**Methods:**

Schedule: 12h-timed-flat-infusion 5-FU 700-1000 mg/m^2^/d 1-2, 8-9, 15-16, 22-23, with 100 mg/m^2^/d increase for dose level; DTX 50 mg/m^2^ d 1, 15 fixed dose, OXP at three increasing dose-levels 60-70-80 mg/m^2^ d 8, 22, every 4 weeks. Intra- and inter-patients dose-escalation was planned.

**Results:**

Ten fit <75 years patients were enrolled: median age 59; young-elderly 4 (40%). From first to fifth dose level, 5 patients (1 per cohort) were enrolled according to intra-patient dose escalation, no dose-limiting toxicity (DLT) were reported. At sixth level, 1 DLT, G2 diarrhea, was reported, thus other 2 patients were enrolled, DLT 1/3 patients (33%). Maximum tolerated dose (MTD) was not reached. 5-FU and OXP recommended doses (RD) were 1000 mg/m^2^/d and 80 mg/m^2^, respectively. To confirm RD, other 3 patients were enrolled, without DLT. Cumulative G3-4 toxicities were: neutropenia 50%, leucopenia 20%, hypoalbuminemia 10%, mucositis 10%, asthenia 20%. Limiting toxicity syndromes were 30%, 25% in young-elderly, all multiple site. Objective response rate intent-to-treat 60%, disease control rate 90%. After 15 months follow-up, progression-free and overall survival, 6 and 17 months, respectively.

**Conclusions:**

First line intensive FD/FOx regimen adding DXT/5-FU/OXP can be safely administered at recommended doses in advanced GC, with promising high activity and efficacy.

## INTRODUCTION

Clinical management of advanced gastric cancer (GC) faces with different options of medical treatment strategies according to patients’ fitness (age, performance status (PS), comorbidities), extension of primary tumor and related symptoms, influencing nutritional and functional conditions. Over the last 40 years, different active drugs were evaluated, and used in mono, doublet, and triplet chemotherapy combinations, improving activity, quality of life (QoL), and overall survival (OS). 5-fluorouracil (5FU)-based regimens consisting of mono, doublet or triplet combinations of chemotherapeutic drugs, improved activity and efficacy of first line advanced or metastatic GC.

5-FU, doxorubicin plus mitomycin (FAM) and 5-FU plus doxorubicin (Adriamycin) (FA) did not significantly increased OS vs 5-FU alone [1]. Epirubicin, cisplatin, and 5-FU (ECF) vs FAMTX significantly improved survival and QoL: overall response rate (ORR) 45 vs 21%, progression-free survival (PFS) 7.4 vs 3.4 months, OS 8.9 vs 5.7 months [2]. Mitomycin, cisplatin, and protracted venous-infusion (PVI) 5-FU, MCF regimen, compared with ECF, did not significantly increased outcomes: ORR 44.1 vs 42.4%, PFS 7 months for both regimens, OS 8.7 vs 9.4 months; QoL was better with ECF [3].

Doublet 5-FU leucovorin/OXP (FLO) reported ORR 44.9%, PFS 6.2 months, OS 8.6 months [4]. A trend toward improved PFS 5.8 vs 3.9 months, with no significantly different OS (10.7 vs 8.8 months), was reported with FLO association. [5]. In elderly >65 years patients, FLO resulted in significantly increased ORR (41.3 vs 16.7%), time to treatment failure (5.4 vs 2.3 months), PFS (6.0 vs 3.1 months), and improved OS (13.9 vs 7.2 months).

In REAL2 trial, patients received triplet with epirubicin and cisplatin plus either 5-FU (ECF) or capecitabine (ECX) or triplet with epirubicin and OXP plus either 5-FU (EOF) or capecitabine (EOX) [6]: OS were 9.9, 9.9, 9.3, 11.2 months, respectively, significantly longer with EOX vs ECF; PFS and ORR not significantly different. In ML17032 trial, cisplatin plus capecitabine (XP) compared to cisplatin plus 5-FU (FP) demonstrated: ORR 41 vs 29%, PFS 5.6 vs 5.0 months, OS 10.5 vs 9.3 months [7]. OS and ORR were superior with capecitabine- vs 5-FU-based combinations [8], PFS not significantly different in a meta-analysis of these two randomized phase III trials.

Major challenge of more drugs addition in a chemotherapy regimen is designing proper schedule and doses, providing the adequate balance between dose intensity (DI) of each drug and safety. DTX addiction to cisplatin/5-FU-based triplet chemotherapy regimen was evaluated in randomized studies [9, 10], at different doses and schedules, with significantly increased toxicity, limiting the favourable impact and expected efficacy of the association. In phase II/III V325 trial, DCF significantly increased outcomes compared with CF [9]: ORR 37 vs 25%, *P* 0.01, PFS 5.6 vs 3.7 months, *P* < 0.001, and OS 9.2 vs 8.6 months, *P* 0.02.

Modulated DCF regimen demonstrated efficacy and improved safety profile in different clinical trials [11–15]. FLOT schedule reported in a phase II trial ORR 57.7%, PFS 5.2 months, OS 11.1 months [11]. Outcomes were not significantly different with DCF compared with modified DCF (mDCF) in a retrospective evaluation [12]: ORR 45.6 vs 46.7%, *P* > 0.05, PFS and OS 7.4 vs 6.5, 9.9 vs 8.6 months *P* > 0.05. In a phase II randomized trial, DTX/OXP/5-FU reported higher ORR and longer PFS and OS compared with DTX/OXP, and DTX/OXP/capecitabine: 46.6%, 7.66 months, 14.59 months [14]. First line DOC triplet association [13], reported ORR 52.1%, PFS and OS 6.9 and 12.6 months, respectively. In another randomized phase II study, mDCF regimen improved efficacy compared with DCF [15]: 6-month PFS 63 vs 53%, median OS 18.8 vs 12.6 months, *P* 0.007.

More, different clinical trials evaluated irinotecan (CPT-11) in advanced GC. In a phase III randomized trial, CPT-11/5-FU was not inferior to cisplatin/5-FU, OS 9.0 vs 8.7 months, ORR 31.8 vs 25.8% [16]. In a randomized phase II trial, capecitabine was added to CPT-11 or cisplatin: ORR 37.7 vs 42.0%, PFS 4.2 vs 4.8 months, with trend toward better OS 10.2 vs 7.9 months [17].

In French randomized trail comparing first line epirubicin, cisplatin, and capecitabine (ECX) with 5-FU, leucovorin, and CPT-11 (FOLFIRI), with predefined second line (FOLFIRI for ECX, ECX for FOLFIRI arm), time to failure was significantly longer with FOLFIRI than ECX (5.1 vs 4.2 months, *P* 0.008) [18].

More recently, in phase III randomized SPIRITS trial, S-1 plus cisplatin was compared with S-1 [19]. OS and PFS were significantly longer in S-1/cisplatin, 13.0 vs 11.0 months, *P* 0.04, and 6.0 vs 4.0 months, *P* <0.0001. In western population, cisplatin and S-1 determined ORR 51%, median OS 10.9 months [20]. In phase III First-Line Advanced Gastric Cancer Study (FLAGS), S-1 plus cisplatin resulted in similar OS compared with 5-FU plus cisplatin: 8.6 vs 7.9 months [21, 22]. In a randomized phase 3 trial, OS was statistically non-inferior with S-1/cisplatin compared to 5-FU/cisplatin (8.6 vs 7.9 months) [23].

We previously developed, in metastatic colorectal cancer setting, clinical trials proposing the concept of alternating chemotherapy administration schedules, as well as 12h-timed-flat-infusion (TFI)/5-FU administration modality, to optimize safety profile and maintain adequate dose intensity of each drug, in order to intensify first line regimens in fit patients [24–29]. The present dose-finding study, proposing intensive triplet chemotherapy combining first line DXT/OXP/5-FU in metastatic GC patients, assess OXP and 5-FU doses to be recommended in association with DXT for prospective clinical trials, safety and preliminary activity.

## RESULTS

### Patient demographics

Ten consecutive patients were enrolled (Table 1): Male/Female ratio, 6/4; median age, 59 years; 4 (40%) young-elderly (≥ 65 <75 y); CIRS stage primary 6 (60%), intermediate 4 (40%) [30]; WHO PS 0 8 (80%), 1-2 2 (20%); primary tumor location, antrum 2 (20%), body 6 (60%), fundus 1 (10%), cardiac 1 (10%). Metastatic disease synchronous in all patients. Metastatic sites: liver 7 patients (70%), lung 4 (40%), lymph nodes 4 (40%), local recurrence 7 (70%), peritoneal carcinomatosis 3 (30%). Metastatic site was single in 1 patient (10%) with liver metastates, multiple in 9 patients (90%). Liver metastases were all multiple.

**Table 1:** Patients’ features

No. of patients	Total N. (%)
	10
Sex	
Male/Female	6/4
Age, years	
median	59
range	42-73
≥ 65 years	4 (40)
CIRS stage	
primary	6 (60)
intermediate	4 (40)
Secondary	-
WHO Performance Status	
0	8 (80)
1-2	2 (20)
Metastatic disease	
synchronous	10 (100)
metachronous	-
Primary tumor location	
antrum	2 (20)
body	6 (60)
fundus	1 (10)
cardias	1 (10)
No. of involved sites	
1	1 (10)
≥ 2	9 (90)
Sites of metastases	
liver	7 (70)
lung	4 (40)
lymph nodes	4 (40)
local recurrence	7 (70)
peritoneal carcinomatosis	3 (30)

Abbreviation: CIRS, Cumulative Illness Rating Scale; WHO, World Health Organization.

### Dose finding

DTX was administered at 50 mg/m^2^ fixed dose (Table 2). At the first dose-level (5-FU 700 mg/m^2^/d and OXP 60 mg/m^2^), 1 patient was enrolled and 2 cycles of treatment were administered; no dose-limiting toxicity (DLT) was observed. At the second dose level (5-FU 800 mg/m^2^/d and OXP 60 mg/m^2^), 2 patients (1 new patient) were treated, and 4 cycles of treatment were administered; no DLT was observed. At the third dose level (5-FU 900 mg/m^2^/d and OXP 60 mg/m^2^), 3 patients (1 new patient) were treated, and 5 cycles of treatment were administered; no DLT was observed. At the fourth dose level (5-FU 900 mg/m^2^/d and OXP 70 mg/m^2^), 3 patients (1 new patient) were treated, and 4 cycles of treatment were administered; no DLT was observed. At the fifth dose level (5-FU 900 mg/m^2^/d and OXP 80 mg/m^2^), 6 patients (1 new patient) were treated, and 9 cycles of treatment were administered; no DLT was observed. At sixth dose level (5-FU 1000 mg/m^2^/d and OXP 80 mg/m^2^), 9 patients (5 new patients) were treated, and 22 cycles were administered. A DLT, G2 diarrhea, was observed in 1 patient (no new patient), then other 2 patients were enrolled at this dose level without other DLT, 1 out of 3 patients (33%) of the first cohort. Thus, maximum tolerated dose (MTD) was not reached, and the recommended doses of the association was 12h-TFI/5-FU 1000 mg/m^2^/d d1-2, 8-9,15-16, 22-23; DTX 50 mg/m^2^ d1, 15; OXP 80 mg/m^2^ d8, 22. In order to confirm the recommended doses, other 3 patients were enrolled at this dose-level, without other DLT. Thus, 1 DLT was observed out of 9 patients (11%) and out of 22 cycles of treatment (4%).

**Table 2:** 5-fluorouracil and oxaliplatin dose-finding

Dose levels	docetaxel (mg/m^2^ d1,15)- oxaliplatin (mg/m^2^ d8,22) 5-fluorouracil (mg/m^2^/d d1-2,8-9,15-16,22-23)	No. patients (new patients)^a^	No. cycles	No. patients with DLT/ total patients (%)	No. patients with DLT/ new patients (%)	No. cycles with DLT/ total cycles (%)	DLTs
I	50-60-700	1 (1)	2	0/1 (0)	0/1 (0)	0/2 (0)	-
II	50-60-800	2 (1)	4	0/2 (0)	0/1 (0)	0/4 (0)	-
III	50-60-900	3 (1)	5	0/3 (0)	0/1 (0)	0/5 (0)	-
IV	50-70-900	3 (1)	4	0/3 (0)	0/1 (0)	0/4 (0)	-
V	50-80-900	6 (1)	9	0/6 (0)	0/1 (0)	0/9 (0)	-
VI	50-80-1000	9 (5)	22	1/9 (11)	0/5 (0)	1/22 (4)	G2 diarrhea

^a^ intra- and inter-patients dose escalation.

Abbreviation: DLT, dose-limiting toxicity.

### Dose-intensity

Median number of administered cycles of treatment was 5.5 (range 3-8). Median received dose intensity (rDI) per patient were: DTX 22.75 mg/m^2^/week (91% of recommended-DI), OXP 33.5 mg/m^2^/week (83.75%), 5-FU 1657.5 mg/m^2^/week (82.87%), respectively. In 4 young-elderly patients, median rDIs per patient were: DTX 21.25 mg/m^2^/week (85% of recommended-DI), OXP 29.5 mg/m^2^/week (73.75%), 5-FU 1513.75 mg/m^2^/week (75.68%), respectively.

### Toxicity

Table 3 describes cumulative toxicities in 10 enrolled patients and in 50 administered cycles. No patient discontinued treatment due to limiting toxicity. Cumulative G3-4 toxicities, by patients were: neutropenia 50%, leucopenia 20%, hypoalbuminemia 10%, mucositis 10%, asthenia 20%. Limiting toxicities were reported in 1 out of 4 (25%) young-elderly patients: G3 hypoalbuminemia, G3 mucositis. G2 toxicities by patients were: diarrhea 40%, asthenia 60%, hypoalbuminemia 30%, neurotoxicity 30%, anemia 10%, thrombocytopenia 10%. No case of thrombosis, hemorrhage/bleeding, cardiac or cerebrovascular ischemia, G3-4 thrombocytopenia, or toxic deaths were observed.

**Table 3:** Cumulative toxicity

Number	Patients				Cycles			
	10				50			
*NCI-CTC Grade*	1	2	3	4	1	2	3	4
Nausea (%)	5 (50)	1 (10)	-	-	11 (22)	1 (2)	-	-
Vomiting (%)	1 (10)	1 (10)	-	-	2 (4)	1 (2)	-	-
Diarrhea (%)	4 (40)	4 (40)	-	-	12 (24)	5 (10)	-	-
Hypoalbuminemia (%)	-	3 (30)	1 (10)	-	-	4 (8)	1 (2)	-
Constipation (%)	4 (40)	2 (20)	-	-	9 (18)	2 (4)	-	-
Mucositis (%)	4 (40)	1 (10)	1 (10)	-	8 (16)	2 (4)	1 (2)	-
Anorexia (%)	2 (20)	3 (30)	-	-	6 (12)	3 (6)	-	-
Asthenia (%)	2 (20)	6 (60)	2 (20)	-	16 (32)	13 (26)	2 (4)	-
Neurotoxicity (%)	7 (70)	3 (30)	-	-	31 (62)	4 (8)	-	-
Hypertension (%)	-	-	-	-	-	-	-	-
Hypotension (%)	-	-	-	-	-	-	-	-
Hematuria (%)	-	-	-	-	-	-	-	-
Gengival recession/gengivitis (%)	-	-	-	-	-	-	-	-
Rhinitis (%)	1 (10)	1 (10)	-	-	5 (10)	1 (2)	-	-
Epistaxis (%)	2 (20)	-	-	-	4 (8)	-	-	-
Hand-foot skin reaction (%)	2 (20)	-	-	-	2 (4)	-	-	-
Hypokalemia (%)	2 (20)	-	-	-	3 (6)	-	-	-
Hypertransaminasemy (%)	2 (20)	-	-	-	2 (4)	-	-	-
Hyperpigmentation (%)	2 (20)	-	-	-	2 (4)	-	-	-
Fever without infection (%)	-	-	-	-	-	-	-	-
Alopecia (%)	2 (20)	3 (30)	-	-	7 (14)	3 (6)	-	-
Anemia (%)	4 (40)	1 (10)	-	-	5 (10)	1 (2)	-	-
Leucopenia (%)	2 (20)	3 (30)	1 (10)	1 (10)	7 (14)	8 (16)	1 (2)	1 (2)
Neutropenia (%)	-	3 (30)	4 (40)	1 (10)	4 (8)	9 (18)	6 (12)	3 (6)
Thrombocytopenia (%)	-	1 (10)	-	-	-	2 (4)	-	-

Abbreviation: NCI-CTC, National Cancer Institute Common Toxicity Criteria.

Overall, limiting toxicity syndromes (LTS) [25, 28], all multiple sites (LTS-ms), were observed in 3 patients (30%): 1 out of 4 young-elderly patients (25%), characterized by G3 hypoalbuminemia, G3 mucositis, and G2 neurotoxicity, associated with G2 asthenia, G3 neutropenia; 2 out of 6 non-elderly patients (33.3%), characterized by G3 asthenia, G2 neurotoxicity and G4 leuconeutropenia, associated with G2 diarrhea, G2 anorexia, G2 alopecia, in 1 patient, and by G3 asthenia and G2 neurotoxicity, associated with G2 constipation, G2 diarrhea, G2 mucositis, G3 neutropenia, in 1 patient.

### Activity and efficacy

Overall, 10 patients were enrolled (Table 4), 7 treated in the dose escalation phase and 3 at the recommended doses. In the intent-to-treat and as-treated analyses all 10 patients were evaluable: ORR was 60% (α 0.05, CI ± 32). We observed 1 complete and 5 partial responses, 3 stable disease and 1 progression. Disease Control Rate (DCR) was 90% (α 0.05, CI ± 19). After a median follow-up of 15 months, median PFS was 6 months (3-15): 9 events occurred. Median OS was 17 months (5+ - 26): 7 events occurred. Secondary resection of liver metastasis was performed in 1 patient with PFS of 10 months, and clinical complete response after triplet chemotherapy regimen.

**Table 4:** Activity and efficacy data

	Intent-to-treat Analysis		As-treated Analysis	
	No	%	No	%
**Enrolled patients**	10	100	10	100
**Evaluable patients**	10	100	10	100
**Objective Response**	6	60 (CI ± 32)	6	60 (CI ± 32)
Partial Response	5	50	5	50
Complete Response	1	10	1	10
**Stable Disease**	3	30	3	30
**Progressive Disease**	1	10	1	10
**Median Progression-free survival, months**	6	90		
Range	3-15			
Progression events	9			
**Median Overall Survival, months**	17	70		
Range	5+-26			
Deaths	7			

Second line treatments were CPT-11 or ramucirumab-based chemotherapy associations in suitable patients.

## DISCUSSION

One of the major reason that can justify the failure of the association of DTX to doublet platinum/5-FU to significantly increase activity and efficacy of metastatic GC patients in clinical practice can be ascribed to toxicity limiting the effective realization of this intensive first line chemotherapeutic strategy. The present dose-finding study proposing DTX, 5-FU and OXP triplet chemotherapy association, recommended 5-FU dose 1000 mg/m^2^/d and OXP 80 mg/m^2^ for safely administration in clinical trials to properly evaluate their contribution to clinical outcome as first line treatment in metastatic GC patients. FD/FOx association was feasible at median DTX rDI 92% (23 mg/m^2^/week), 5-FU rDI 83% (1657 mg/m^2^/week), and OXP rDI 92% (23 mg/m^2^/week). Cumulative G3-4 toxicities were represented by neutropenia 50%, leucopenia 20%, hypoalbuminemia 10%, mucositis 10%, asthenia 20%. Three individual LTS, all LTS-ms, were reported in 30% patients. G2 toxicities were represented by diarrhea 40%, asthenia 60%, hypoalbuminemia 30%, neurotoxicity 30%, anemia 10%, thrombocytopenia 10%.

Major features of the proposed schedule are: 12 hours nightly-TFI infusion 5-FU limiting FU-related side effects, specifically mucositis and diarrhea, compared to all combinations containing 5-FU, and weekly alternating infusion of DTX and OXP, modulating intensity of the concomitant infusion of triplet drugs every 3 weeks. To this concern, it may limit specifically reported toxicity: mucositis and diarrhea related to 5-FU alone; anorexia, nausea, vomiting, leucopenia, thrombocytopenia with FAM [1]; haematological with FAMTX [2]; G3-4 neutropenia with ECF, and thrombocytopenia and hand-foot syndrome with MCF [3]. Grade 3-4 toxicities with FLO regimen were: neutropenia 38%, thrombocytopenia 4%, anemia 11%, neurotoxicity 21% [4]. Cumulative toxicity reported with FLO regimen, 24h infusion 5-FU 2600 mg/m^2^, leucovorin 200 mg/m^2^, and OXP 85 mg/m^2^, compared with 24h infusion 5-FU 2000 mg/m^2^, leucovorin 200 mg/m^2^, and cisplatin 50 mg/m^2^, every 2 weeks, was favourable: anemia (54 vs 72%), nausea (53 vs 70%), vomiting (31 vs 52%), alopecia (22 vs 39%), asthenia (19 vs 34%), neurotoxicity (22 vs 63%) [5]. In REAL2 trial, safety profile of capecitabine and 5-FU were similar; lower incidences of G3-4 neutropenia, alopecia, renal toxicity, but slightly higher incidences of diarrhea and neuropathy were associated with OXP compared with cisplatin [6]. In ML17032 trial, prevalent G3-4 toxicities in XP vs FP were: neutropenia (16 vs 19%), vomiting (7 vs 8%), stomatitis (2 vs 6%) [7].

Safety of DTX addiction to cisplatin/5-FU in a triplet chemotherapy regimen was evaluated in different clinical studies [9, 10].

Our preliminary data concerning activity and efficacy show that the present schedule of safely administration of DTX/5-FU/OXP association may achieve 60% ORR, 90% DCR, median PFS 6 months, median OS 17 months. ECF regimens reported ORR 44.1-45%, PFS 7-7.4 months, OS 8.7-8.9 months [2, 3]; FLO association reported ORR 44.9%, PFS 5.8-6.2 months, OS 8.6 months [4, 5], particularly relevant in elderly patients, ORR 41.3%, PFS 6.0 months, OS 13.9 months [5]; EOX regimen reached 11.2 months OS [7]. In fit patients, intensive regimens adding DTX to cisplatin/5-FU reported ORR ranging between 37-46.7%, PFS 5.6-9.2 months, OS 8.6-12.6 months [5, 9, 12, 15]. In V325 trial, patients were randomized to DXT 75 mg/m^2^ and cisplatin 75 mg/m^2^ d1 plus 5-FU 750 mg/m^2^/d d1-5, every 3 weeks (DCF) or cisplatin 100 mg/m^2^ d1 plus 5-FU 1000 mg/m^2^/d d1-5, every 4 weeks (CF) [9]. Prevalent G3-4 toxicities in DCF vs CF arms were: neutropenia (82 vs 57%), stomatitis (21 vs 27%), diarrhea (19 vs 8%), asthenia (19 vs 14%).

Different clinical trials evaluated modulated DCF regimen and demonstrated improved safety profile [11–15]. Modified DCF regimen reported ORR 45.6%, PFS 7.4 months, OS 9.9-18.8 months [12, 15]. In a retrospective evaluation DCF, 75 mg/m^2^ DTX and cisplatin d1 plus 5-FU 750 mg/m^2^/day d1-5, every 3 weeks, was compared with modified DCF (mDCF), including 60 mg/m^2^ DTX and cisplatin d1 and 5-FU 600 mg/m^2^ continuous infusion d1-5, every 3 weeks [12]. Prevalent G3-4 toxicities were higher in DCF arm: neutropenia (48.2 vs 13.6% *P* 0.003), anemia (21.2 vs 4.5% *P* 0.06), nausea (44.7 vs 13.6% *P* 0.008), and vomiting (31.8 vs 4.5% *P* 0.01). mDCF regimen (48-h infusion 5-FU 2000 mg/m^2^, DTX 40 mg/m^2^ d1, cisplatin 40 mg/m^2^ d3, every 2 weeks) compared with DCF (DXT 75 mg/m^2^, cisplatin 75 mg/m^2^, and 5-FU 750 mg/m^2^ IV over 5 days with granulocyte colony-stimulating factor, every 3 weeks) [15], reported 76% G3-4 toxicities in mDCF arm; DCF arm closed early because of 90% limiting toxicities.

Addition of DTX to OXP and 5-FU- or capecitabine-based first line chemotherapy represents a step forward to intensify medical treatment of GC patients, maintaining good tolerability: ORR 46.6-57.7%, PFS 5.2-7.6 months, OS 11.1-14.5 months [11, 14]. Prevalent G3-4 toxicities reported with FLOT schedule, biweekly DTX 50 mg/m^2^, OXP 85 mg/m^2^, and 24-h infusion 5-FU 2600 mg/m^2^ plus leucovorin 200 mg/m^2^, were: neutropenia 48.1%, leucopenia 27.8%, diarrhea 14.8%, asthenia 11.1% [11]. Better safety profile was reported with DTX/OXP/5-FU association [14]: G3-4 adverse events 25 vs 37 vs 38%; febrile neutropenia 2, 14, and 9%, respectively. Common G3-4 toxicities were: fatigue 21%, neurotoxicity 14%, diarrhea 13%.

Neutropenia was the most common G3-4 toxicity (41%) with DOC triplet association, DTX 60 mg/m^2^, OXP 100 mg/m^2^ d1, and capecitabine 500 mg/m^2^/bidie, every 3 weeks, reaching ORR 52.1%, PFS 6.9 months, OS 12.6 months [13].

More, different clinical trials evaluated CPT-11 in advanced GC. CPT-11/5-FU was associated with a better safety profile, less treatment discontinuation rate (10.0 vs 21.5%), less frequent neutropenia, thrombocytopenia and mucositis, but not diarrhea [16]. In a randomized phase II trial, capecitabine 1000 mg/m^2^, twice daily for 14 days, was added to CPT-11 250 mg/m^2^ or cisplatin 80 mg/m^2^ d1, every 3 weeks: prevalent G3-4 toxicities in platinum regimen were thrombocytopenia (18.2 vs 1.8%), nausea (23.6 vs12.3%) and vomiting (16.4 vs 1.8%), in CPT-11 arm diarrhea (22.8 vs 7.3%) [17]. In French trail, FOLFIRI was better tolerated than ECX (overall G3-3 toxicity rate, 69 vs 84%; P < 0.001) [18].

More recently, in the SPIRITS trial, S-1 (40-60 mg), twice daily for 3 weeks, plus cisplatin 60 mg/m^2^ d8, followed by 2-week rest, was compared with S-1 for 4 weeks, followed by a 2-week rest [19]. G3-4 leucopenia, neutropenia, anemia, nausea, and anorexia, were more frequent in S-1/cisplatin arm. In west population, cisplatin 75 mg/m^2^ d1 and S-1 25 mg/m^2^ bid d1-21, every 28 days, determined G3-4 fatigue (26%), neutropenia (26%), vomiting (17%), diarrhea (15%), nausea (15%) [20]. In FLAGS trial, S-1 50 mg/m^2^ d1-21 plus cisplatin 75 mg/m^2^ d 1, every 28 days, more frequent G3-4 toxicities included asthenia (24%), vomiting (17%), nausea (15%), diarrhea (13%), and neutropenia (19%) [21, 22]. Less frequent G3-4 toxicities were observed in cisplatin/S-1 compared with cisplatin/5-FU arm: neutropenia (32.3 vs 63.6%), stomatitis (1.3 vs 13.6%), hypokalemia (3.6 vs 10.8%). Statistically significant safety advantages were also observed with S-1: G3-4 neutropenia 18.6 vs 40.0%, febrile neutropenia 1.7 vs 6.9%, mucositis 1.3 vs 13.6% [23].

The consideration of the present schedule of triplet chemotherapy may also represent a model for integration of different antimetabolite (capecitabine, S-1) and/or different drugs (irinotecan) into the same triplet schedule. Thus, the definition of the proper schedule to safely add DTX to OXP/5-FU doublet chemotherapy represents a mainstay to further investigate in randomized clinical studies whether more intensive first line triplet medical treatments could increase clinical outcome in metastatic GC patients.

Trastuzumab addiction to capecitabine/cisplatin or 5-FU/cisplatin in HER2 positive patients significantly improved OS up to 16 vs 11.8 months in immunohistochemistry (IHC) 2+ and fluorescence *in-situ* hybridization (FISH) positive or IHC3+ [31]. Most common adverse events were: nausea (67 vs 63%), vomiting (50 vs 46%), neutropenia (53 vs 57%), cardiac adverse events (6% both arms). Definition of a feasible and safe triplet chemotherapy schedule could be potentially developed for a more intensive first line regimen adding trastuzumab in HER2 positive metastatic GC patients.

Second line CPT-11 plus mitomycin-c reported ORR 32%, PFS 4 months, OS 8 months [32]. PFS and OS were 3.1 and 6.5 months with capecitabine and CPT-11 [33]. Retrospective evaluation of DTX plus CPT-11, after platinum-based therapy reported PFS 11 weeks, OS 24 weeks [34]. In a phase III study, among patients refractory to fluoropyrimidine plus platinum, ORR, PFS and OS were not significantly different in paclitaxel compared to CPT-11: 20.9%, 3.6 and 8.4 months vs 13.6%, 2.3 and 9.5 months, respectively [35]. FOLFIRI in patients who failed DTX-containing first-line therapy reached ORR 22.8%, PFS 3.8 months and OS 6.2 months [36].

In metastatic GC patients, progressed after first-line platinum- or fluoropyrimidine-containing chemotherapy, OS was significantly increased to 5.2 vs 3.8 months *P* 0.047, with ramucirumab, 8 mg/kg every 2 weeks, compared with placebo [37]. Addiction of ramucirumab to paclitaxel, after first-line platinum plus fluoropyrimidine with or without anthracycline, significantly increased OS up to 9.6 vs. 7.4 months, *P* 0.017 [38]. The increasing efficacy of ramucirumab plus taxane as second line treatment point the discussion on the possibility to adopt a sequential treatment strategy alternative to a more intensive first line regimen in metastatic GC, particularly in patients unfit for such an intensive combined therapy, due to clinical parameters, age, comorbidity, functional/nutritional conditions.

## MATERIALS AND METHODS

### Patient eligibility

Patients were eligible if they had histologically confirmed diagnosis of measurable metastatic GC; age 18-75 years; World Health Organization (WHO) PS ≤ 2; adequate hematological, renal and hepatic functions; life expectancy more than 3 months.

CIRS was used to evaluate the comorbidity status, and only patients with primary and intermediate CIRS stage were enrolled [30]. Primary CIRS stage consisted of: independent Instrumental Activity of Daily Living (IADL), and absent or mild grade comorbidities; intermediate CIRS stage consisted of dependent or independent IADL, and < 3 mild or moderate grade comorbidities. Patients with secondary CIRS stage, consisting of ≥ 3 comorbidities or a severe comorbidity, with or without dependent IADL, were not enrolled. Criteria to define patients unfit for the proposed treatment strategy were: uncontrolled severe diseases; cardiovascular disease (uncontrolled hypertension, uncontrolled arrhythmia, ischemic cardiac diseases in the last year); thromboembolic disease, coagulopathy, preexisting bleeding diatheses.

First line DTX association to 5-FU and OXP, was proposed to consecutive eligible metastatic GC patients as a treatment strategy in clinical practice, chosen among those in indication and approved by Agenzia Italiana del Farmaco (AIFA) for administration *in label* in Italian public hospitals, and published in Gazzetta Ufficiale Repubblica Italiana (“Elenco dei Medicinali erogabili a totale carico del Servizio Sanitario Nazionale”, Gazzetta Ufficiale Repubblica Italiana N.1, 2 Gennaio 2009). Thus, it was not necessary any approval by ethics committee and institutional review board, because patients were treated with conventional treatments without any additional medical intervention out of the best common clinical practice. All patients provided written, informed consent to the proposed *in label* treatment strategy. Treatment was conducted in accordance with the Declaration of Helsinki.

### Methods

### Schedule

It was a dose-finding study evaluating safety and activity of triplet chemotherapy association, consisting of DTX, 5-FU and OXP, as first line treatment in advanced GC patients. Triplet chemotherapy association was administered according to the following schedule: DTX (Taxotere; Sanofi-Aventis, Milan, Italy), administered over 60 minutes as intravenous infusion in 250 ml of NaCl 0.9%, at the dose of 50 mg/m^2^ d1, 15; TFI/5-FU (Fluorouracil Teva^®^, Teva), 700-800-900-1000 mg/m^2^/die, over 12-hour (from 10:00 p.m to 10:00 a.m.), d1-2, 8-9, 15-16 and 22-23; OXP (Eloxatin; Sanofi-Aventis, Milan, Italy), over 2-hours as an intravenous infusion in 250 ml of dextrose 5%, at the dose of 60-70-80 mg/m^2^ d8, 22. Cycles repeated every 4 weeks. 5-FU was administered by a portable pump (CADD Plus, SEVIT) using a venous access device.

### Study design

Physical examination and routine laboratory tests were performed at baseline and every week on-treatment, including complete blood cell count, electrolytes, liver and renal function, urine examination and coagulation function; tumor markers every 4 weeks; electrocardiogram every cycle and echocardiogram at baseline, and every 3 cycles of treatment.

Primary end-point was to define the recommended 5-FU and OXP doses. Secondary end-points were evaluation of toxicity, ORR, PFS, OS. Toxicity was registered every week according to National Cancer Institute Common Toxicity Criteria (version 3.0). DLT was defined: in the intra-patient step, as G2 non-haematological or G3 haematological toxicity; in the inter-patient step, as grade 3-4 non-haematological toxicity (mainly represented by diarrhea, mucositis, neurotoxicity, hand-foot syndrome, asthenia), grade 4 hematologic toxicity (neutropenia), febrile neutropenia, grade 3-4 thrombocytopenia, or any toxicity determining > 2 weeks treatment delay.

To discriminate individual safety, LTS, consisting of at least a limiting toxicity (LT) associated or not to other limiting or G2 toxicities, were evaluated, as previously reported [25, 28]. LTS were classified as LTS single site (LTS-ss), characterized only by the LT, and LTS multiple sites (LTS-ms), ≥ 2 LTs or a LT associated to other, at least G2, non-limiting toxicities.

ORR was evaluated according to RECIST criteria [39]; pathologic complete response was defined as absence of residual cancer cells in surgically resected specimens; PFS and OS, using Kaplan-Meier method [40]. PFS was defined as length of time between the beginning of treatment and disease progression or death (resulting from any cause) or to last contact; OS as length of time between the beginning of treatment and death or to last contact.

Patients were evaluated at baseline and after treatment by a multidisciplinary team, consisting of medical oncologist, radiotherapist, surgeon, and radiologist, to dynamically evaluate multimodality treatment strategy. Surgical resection was defined R0, if radical surgery, R1, if microscopic residual cancer cells were present at resection margins. Surgery was recommended > 4-6 weeks after chemotherapy discontinuation.

Clinical evaluation of response was planned by CT-scan; PET and MRI were added based on investigators’ assessment; response on primary gastric tumor was also evaluated by endoscopy. Follow-up was scheduled every three months up to disease progression or death.

### Statistical design

This dose-finding study was developed to verify recommended OXP dose, by 3 escalating dose steps at 60, 70 and 80 mg/m^2^, and 5-FU dose, by 4 escalating dose steps at 700, 800, 900 and 1000 mg/m^2^/d, according to an intra- and inter-patient approach [41, 42]. It is preliminary to a phase II study, evaluating activity and efficacy of triplet chemotherapy association, assuming as minimal interesting activity a rate of 40%, according to Simon two stage design [42].

## CONCLUSIONS

The present dose-finding study proposed a feasible and safe schedule of triplet DTX/5-FU/OXP association, FD/FOx regimen, at 5-FU and OXP recommended doses 1000 mg/m^2^/d and 80 mg/m^2^, respectively, that should be evaluated in prospective trials as first line treatment in metastatic GC patients.

## Abbreviations

CIRS: Cumulative Illness Rating Scale
CF: cisplatin, 5-fluorouracil
CPT-11: irinotecan
DCF: docetaxel, cisplatin, 5-fluorouracil
DCR: disease control rate
DI: dose intensity
DLT: dose-limiting toxicity
DTX: docetaxel
ECF: epirubicin, cisplatin, 5-fluorouracil
ECX: epirubicin, cisplatin, capecitabine
EOF: epirubicin, oxaliplatin, 5-fluorouracil
EOX: epirubicin, oxaiplatin, capecitabine
FAM: 5-FU, doxorubicin, mitomycin
FLO: 5-FU, leucovorin, oxaliplatin
FOLFIRI: 5-fluorouracil, leucovorin, irinotecan
FP: cisplatin, 5-fluorouracil
GC: gastric cancer
IADL: Instrumental Activity of Daily Living
LTS: limiting toxicity syndromes
LTS-ms: limiting toxicity syndromes multiple sites
MCF: mitomycin, cisplatin, 5-fluorouracil
mDCF: modified docetaxel, cisplatin, 5-fluorouracil
MTD: maximum tolerated dose
ORR: objective response rate
OS: overall survival
OXP: oxaliplatin
PFS: progression-free survival
PS: performance status
QoL: quality of life
RD: recommended dose
rDI: received dose intensity
WHO: World Health Organization
XP: cisplatin, capecitabine
5-FU: 5-fluorouracil
